# Wound of the Heart

**Published:** 1829-05

**Authors:** 


### WOUND OF THE HEART.

*Extensive Wound of the Heart, in which the Patient survived one Hour and a Quarter. Necrotomy.* (Winchester County Hospital.)

William Beckett, bricklayer's labourer, ætat. twenty-five, native of Winchester, was brought to the hospital, having been precipitated from a ladder on the top of a house in the vicinity of the town, on some wooden railings, which had perforated the upper part of the abdomen, causing a wound about two inches and a half in extent, through which a vast quantity of the small intestines had protruded. A surgeon being sent for at the moment of the accident, reduced the intestines, applied three sutures to the wound, and directed the man to be conveyed to the hospital. On his arrival he was found to be in a very exhausted state, with great coldness, not only of the extremities, but of the surface of the body generally; the pulse, at the wrist or carotid artery, could not be felt; respiration very laborious and interrupted; extreme jactitation, so much so as to require three or four persons to retain him in bed; complete insensibility; the pupils dilated permanently.

On his admission he was immediately enveloped in warm blankets, bottles of hot water applied to his extremities and stomach, and small quantities of hot brandy-and-water administered every ten minutes, which he swallowed not without considerable difficulty. These endeavours were, however, entirely fruitless: the surface of the body still retained its deathlike coldness, as well as its convulsive movements; the respiration became gradually less frequent and more feeble, and he expired in rather more than an hour and a quarter from the time of the fall.

*Necrotomy.*—The body was examined six hours after death. On tracing the wound, which had been made by the top of the pales on which he had fallen, it was found to have extended through the diaphragm, immediately underneath the sternum, into both ventricles of the heart, and through the septum ventriculorum, leaving a large ragged opening, of one inch in length, in the parietes of each ventricle, by which the blood had escaped from those cavities into the chest, which contained rather more than two quarts of that fluid perfectly uncoagulated. The small intestines in many places were in a complete state of introsusception. With these exceptions, the body presented a most healthy appearance.

*Reflections.*—This case, in one point of view, is highly interesting, and of much practical importance, inasmuch as it clearly manifests that a very considerable injury of the heart may be sustained without causing that immediate dissolution which is commonly supposed uniformly to arise from wounds of this most important and vital organ. We have on record numerous instances of rupture of this viscus from disease, occurring principally, if not entirely, amongst persons at an advanced period of life. In ten patients, who lost their lives from this organic lesion, it was observed eight died instantly, one at the expiration of about two hours, and another at the end of fourteen. The longest period which a person has been known to survive after a wound of the heart is forty-eight hours, exemplified in the case of a soldier on duty at Haslar Hospital, who had a bayonet plunged into the right auricle. On examination after death, it was found that a coagulum had formed, and completely filled up the wound; but, in an effort to evacuate the bowels, a second hemorrhage took place, and he was found dead, sitting on the night chair.*

---

